# Stakeholders’ perspectives on disinvestment of low-value healthcare interventions and practices in Malaysia: an online survey

**DOI:** 10.1017/S0266462324004665

**Published:** 2024-11-15

**Authors:** Hanin Farhana Kamaruzaman, Eleanor Grieve, Ku Nurhasni Ku Abd Rahim, MMG Izzuna, Lee Sit Wai, Erni Zurina Romli, Mohamed Hirman Abdullah, Olivia Wu

**Affiliations:** 1 Health Economics and Health Technology Assessment (HEHTA), School of Health and Wellbeing, University of Glasgow, Glasgow, Scotland; 2 Malaysian Health Technology Assessment Section (MaHTAS), Medical Development Division, Ministry of Health Malaysia, Malaysia, Putrajaya; 3 Hospital Service Development Section, Medical Development Division, Ministry of Health Malaysia, Malaysia, Putrajaya

**Keywords:** disinvestment, stakeholder engagement, health technology reassessment, resource allocation, health care surveys

## Abstract

**Objectives:**

Healthcare disinvestment requires multi-level decision-making, and early stakeholder engagement is essential to facilitate implementation and acceptance. This study aimed to explore the perceptions of Malaysian healthcare stakeholders to disinvestment initiatives as well as identify disinvestment activities in the country.

**Methods:**

A cross-sectional online survey was conducted from February to March 2023 among Malaysian healthcare stakeholders involved in resource allocation and decision-making at various levels of governance. Response frequencies were analyzed descriptively and cross-tabulation was performed for specific questions to compare the responses of different groups of stakeholders. For free-text replies, content analysis was used with each verbatim response examined and assigned a theme.

**Results:**

A total of 153 complete responses were analyzed and approximately 37 percent of participants had prior involvement in disinvestment initiatives. Clinical effectiveness and cost-effectiveness ranked as the most important criteria in assessment for disinvestment. Surprisingly, equity was rated the lowest priority despite its crucial role in healthcare decision-making. Almost 90 percent of the respondents concurred that a formal disinvestment framework is necessary and the importance of training for the program’s successful implementation. Key obstacles to the adoption of disinvestment include insufficient stakeholder support and political will as well as a lack of expertise in executing the process.

**Conclusions:**

While disinvestment is perceived as a priority for efficient resource allocation in Malaysian healthcare, there is a lack of a systematic framework for its implementation. Future research should prioritize methodological analysis in healthcare disinvestment and strategies for integrating equity considerations in evaluating disinvestment candidates.

## 1. Introduction

Disinvestment in healthcare calls for decisions to be made on several different levels ranging from the departmental, organizational, regional, and national levels. Low-value care (LVC), or patient care that provides no or low net benefit in specific clinical scenarios, continues to be one of the most pressing issues in healthcare worldwide, primarily because it increases costs, causes iatrogenic patient harm, and frequently impedes the delivery of high-value care (1). Additionally, the persistence of LVC is attributed to the absence of de-implementation strategies despite major efforts to minimize it over the previous decade (2). In these times of escalating demand for efficiency, there is a need for structured and explicit criteria shaping the disinvestment framework within health care.

Based on our scoping review of published systematic reviews (3), disinvestment programs were predominantly reported mainly in high-income countries, where most of these were championed by health technology assessment (HTA) agencies in that country. However, there are possibilities that informal or small-scale initiatives undertaken by low- and middle-income countries (LMIC) remain unpublished or undiscovered (3). This lack of documentation highlights a significant gap in the literature, underscoring the need for more research focused on disinvestment initiatives in LMICs to better understand their practices, challenges, and outcomes (4).

The implementation phase of disinvestment initiatives presents significant obstacles and complexities in terms of stakeholder engagement, owing primarily to insufficient support, collaboration, and communication (5). A substantial disparity may exist between the way in which experts think disinvestment decisions should be made and how they are actually made at the ground level. This contrast between the technical and political aspects of disinvestment was apparent in the areas of change management, evidence generation, and information sharing (6). Adding to the existing problems, there is a scarcity of information on gathering stakeholder viewpoints on the execution of disinvestment initiatives, with only twelve studies involving healthcare professionals reviewed by Mitchell et al. (7). Thus, it is critical to include key stakeholders in disinvestment at every stage of planning and implementation to secure support and ensure the long-term viability of the initiatives.

Malaysia is classified by the World Bank as an upper-middle-income country and has a well-established dual-tiered system of healthcare services: tax-funded, subsidized government-led public healthcare, and a rapidly expanding private healthcare sector (8, 9). Within its public healthcare system, there are mainly two ways of resource allocation: (i) *top-down or line item budgeting*, in which the financial allocation for healthcare depends on allocation by the central government and is partly based on previous years (10), and (ii) *bottom-up budgeting*, which involves new programs or interventions proposed by departments within the Ministry of Health, usually supported by evidence-based method such as HTA with economic evaluations (9). Meanwhile, the private sectors in Malaysia rely on fee-for-service as the primary method of payment for healthcare facilities (10). The role of HTA in policy formation and decision-making regarding health technologies has become increasingly important and influential over time (8). While Malaysia does not have an explicit benefits package, the effort towards having a formal and well-defined health benefits package in the country is currently ongoing and hence, requires HTA method for its development.

In addressing the gaps in information on disinvestment initiatives with regard to country-specific socioeconomic, geographical distribution, and stakeholder involvement, this survey aimed to describe the perceptions, practices, and receptivity of Malaysian healthcare stakeholders to disinvestment initiatives. The specific objectives of this research include identifying current activities in the Malaysian healthcare system and exploring the important components of implementing disinvestment frameworks from the perspective of key stakeholders in the country.

## 2. Methods

### Study design

This study is part of a mixed-method research project that began with an online survey followed by semi-structured key informant interviews. The survey results are reported in this paper, while the interviews with stakeholders will be published separately.

### Study population and recruitment strategy

Purposive sampling was used to identify survey participants from key stakeholders in Malaysia who may be involved in priority setting and decision-making for resource allocation at various healthcare levels. This includes decision-makers, budget holders, and program managers within the four major Programs in the Ministry of Health (MOH) Malaysia, regional and local leaders such as health state directors, hospital directors, and heads of public health sectors. Participants were also recruited from health care providers, specifically doctors, pharmacists, nurses, and allied health professionals. We also included researchers from local universities offering healthcare courses and research institutes that may be involved in studies related to resource allocation and quality improvement initiatives to obtain a holistic perspective.

Stakeholder representatives were identified from the publicly accessible list of program managers on the Malaysian MOH website (www.moh.gov.my), the list of heads of clinical services in MOH, as well as specific databases for healthcare providers and researchers accessible by MOH personnel. Significant efforts were made to engage potential healthcare professionals involved in resource allocation decision-making with the survey advertised on the Malaysian MOH website, Malaysian Health Technology Assessment Section (MaHTAS) social media, and chain-referral sampling by the experts in HTA and health economics who are part of the MaHTAS Technical Advisory Committee. The initial sample size was 320, based on the identification of stakeholders according to departments and clinical services. Secondary identification of participants was accomplished through the final question in the survey (snowballing), and the survey link was also shared by individuals among their networks. As such, additional samples were identified as the survey progressed.

### Questionnaire development and validation

The questionnaire was developed based on our scoping review (3), published literature related to healthcare disinvestment (11-14), and scoping reviews of theories, frameworks, and models on the de-implementation of LVC (15, 16). There were twenty-three questions with a combination of open-ended questions with free-text responses, closed-ended questions using multiple choice format, sliding scales, as well as a clinical vignette with a ranking-based option. Face validity and pre-testing of the questionnaire were performed by twelve researchers and healthcare professionals from the MOH Malaysia and the University of Glasgow. A content validity index assessment was conducted by six healthcare stakeholders from Malaysia (two program managers, two hospital administrators, a health economist, and a pharmacist) looking into the representativeness, relevancy, and clarity of the questionnaire (Supplementary 1–3).

### Survey design, distribution, and data collection

We designed the online survey using Qualtrics Survey Software (Qualtrics, Provo, UT, USA). The survey questionnaire was structured in five sections: (i) *background information;* (ii) *knowledge and perceptions on disinvestment in healthcare;* (iii) *disinvestment initiatives within organization or workplaces;* (iv) *facilitators and challenges in implementing disinvestment;* (v) *receptivity and expectation on implementation of disinvestment initiatives in Malaysian healthcare system* (Supplementary 4). Participants were asked to suggest any other healthcare stakeholders who could contribute to the study. The survey concluded with an invitation to participate in the follow-up interview that was conducted after the survey closed.

The online survey questionnaire was distributed through emails, the MOH website, and social media platforms between February and March 2023. Reminders were sent two weeks after the first email for those identified on the mailing list. All survey data collection was undertaken using the Qualtrics online platform, and all responses were collected anonymously. To avoid participants taking the survey more than once, the “prevent multiple submission” feature was enabled in the Qualtrics system before distributing the survey.

### Data analysis and reporting

Two investigators (H.F.K. and L.S.W.) independently reviewed all survey responses for clarity and completeness after exporting them from Qualtrics to Excel (Microsoft Corporation). Only completed surveys were included in the final analysis. This is a common practice in survey research that is used to gather the perceptions or opinions of participants, as analyzing incomplete responses may introduce bias and affect the validity of the findings (17). Frequencies of responses were calculated for close-ended questions, and free-text answers were analyzed using content analysis and designated a theme. We then investigated the frequency of each theme to identify the most common perspectives for reporting. We performed cross-tabulation and sub-group analysis for specific questions to compare the responses of different groups of stakeholders. This study was reported in accordance with the Consensus-Based Checklist for Reporting of Survey Studies (CROSS) (18), as outlined in Supplementary 5.

### Ethical considerations

This study was approved by the Medical Research and Ethics Committee, Ministry of Health Malaysia (NMRR-ID-22-02570-6PR-(IIR)) and the Research Ethics Committee, University of Glasgow (200220048). The survey was voluntary and anonymous, and consent was provided by the participants at the start of the online questionnaire.

## 3. Results

### Survey responses

Supplementary 6 presents the survey response flowchart. We issued 341 email invitations to participate in the survey based on the initial identification of key stakeholders in the Malaysian healthcare system, with additional invitations from websites, social media platforms, and snowballing. A total of 461 participants accessed the survey link and consented. However, the majority of these were excluded due to possible ‘bots’ (software that is programmed to do repetitive tasks for users), the completion of only the demographic data in the first section of the survey, and incomplete responses of less than 50 percent of the whole questionnaire. The final analysis included 153 completed surveys after data cleaning.

### Survey respondent characteristics

Table 1 summarizes the characteristics of survey participants. A larger proportion of respondents were from MOH Malaysia, with fifty-two (34 percent) at the headquarters and sixty (39 percent) at medical centers, including MOH hospitals and university hospitals. Around 40 percent of participants were responsible for resource allocation decisions or managing budgets. Fifty-eight respondents (38 percent) were clinical care providers, including medical doctors, clinical pharmacists, nurses, and allied health professionals; and thirty-three were researchers, academics, or experts in HTA or health economics. Survey participants may have many professional responsibilities within the healthcare system; therefore, the primary roles were not restricted to a single category. The participants had diverse levels of experience in the healthcare system, with the majority having less than fifteen years of experience (86 percent), while 14 percent having more than fifteen years of experience in their current roles.

**Table 1.** Characteristics of survey respondents (*N* = 153)

Variables/description		*N* (%)
Workplace		
	MOH Malaysia headquarters	52 (34)
	State Health Department/District	19 (12)
	Hospital/Medical centre (MOH, University hospital)	60 (39)
	Primary health clinic/dental clinic	7 (5)
	Research institute/academia	10 (7)
	Training institute (for nurses, assistant medical officer)	3 (2)
	Not stated	2 (1)
Primary professional role*		
	Resource allocation decision-makers/budget holders	62 (41)
	Clinical care providers	58 (38)
	Researchers/academicians/HTA or health economic experts	33 (22)
	Others (desk officers, medical analysts, regulatory bodies)	23 (15)
Years of experience in current role		
	Less than a year	5 (3)
	1–5 yr	57 (37)
	6–10 yr	42 (28)
	11–15 yr	28 (18)
	More than 15 yr	21 (14)
Level of governance or decision-making*		
	*Single level*	*106 (69)*
	National level	39 (37)
	State/federal territory level	7 (7)
	Regional level (authority/district/region)	12 (11)
	Facility/hospital/organizational level	48 (45)
	*Multiple level*	*47 (31)*
	Involving national level	31 (66)
	At least involving state level	9 (19)
	Regional and facility level	7 (15)
Type of health technologies or scope in the context of decision-making*		
	Pharmaceuticals/drugs	58 (38)
	Non-pharmaceuticals (*e.g., medical devices, digital technologies, surgical and medical procedures, screening and health programmes, diagnostic*)	81 (53)
	Specific fields of care (*e.g., primary care, cancer, public health, food and nutrition)*	83 (54)
	Work force/human resource	47 (31)
	Others (e.g., ICT system, accreditation process, healthcare facilities)	7 (5)

* As participant could select more than one option, it does not sum to 100%.

Abbreviations: MOH, Ministry of Health; HTA, health technology assessment; ICT, information and communications technology.

Approximately 70 percent of individuals had experience in decision-making processes at a single level of governance, comprising 45 percent at the local facility or organization level and 37 percent at the national level. However, 66 percent of those involved in multiple levels of governance were primarily engaged in decision-making at the national level. Over half of the respondents had experience with decision-making related to non-pharmaceutical health technologies, including medical devices, surgical and medical procedures (53 percent), as well as in specific areas of care like public health and primary care (54 percent). Additionally, 38 percent had experience in pharmaceuticals and 31 percent in human resources. Based on the good mix of responses in relation to professional roles, workplace, years of experience in service, and level of governance in resource allocation decision-making, the sample of this study could be considered representative of Malaysian healthcare stakeholders (19).

As shown in Table 1, approximately one-third (37 percent) of the respondents had previous experience with disinvestment or resource reallocation, with 27 activities reported (Supplementary 7). However, beyond quantitative metrics, the complexity of disinvestment decision-making in these activities is compounded by various interrelated components such as financial and budget adjustment (73 percent of the activities reported), affecting patient outcomes and implications in clinical care services (63 percent), as well as the development and enhancement of human resources and specialized skills (34 percent). Supplementary Figure 8 displays additional components associated with these disinvestment activities.

### Understanding the term ‘disinvestment in healthcare’

**Figure 1.**
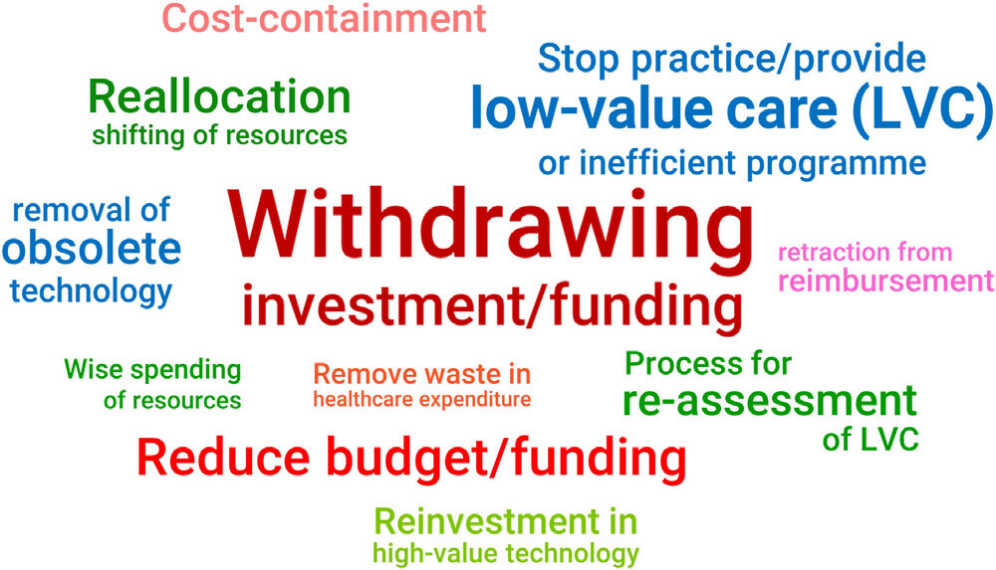
Word cloud for the description of ‘disinvestment in healthcare’.

In the context of Malaysian healthcare, the term ‘disinvestment’ emerges with varied connotations among survey participants (Figure 1 and Supplementary 9). Predominantly, it is characterized as the act of withdrawing investment or funding from healthcare initiatives or programs. This perspective underscores the perception that disinvestment is similar to rationing, which could potentially lead to the cessation of certain practices or services. A significant portion of respondents associates disinvestment with “*the necessity to stop offering LVC and wasteful programs.”* This perspective is consistent with a comprehensive approach to disinvestment, which is a planned redistribution of resources from programs that do not demonstrate clinical effectiveness or provide equivalent value for the investment. Furthermore, some respondents believed that disinvestment involves *“decreasing the budget or funding for health-related programs,”* showing an understanding of the importance of prudent financial management in the healthcare sector. Collectively, these interpretations highlight the diverse aspects of understanding disinvestment in the Malaysian healthcare system while still focusing on its importance in improving resource allocation and increasing the efficiency and efficacy of healthcare services.

### Criteria in conducting assessment for disinvestment

**Figure 2.**
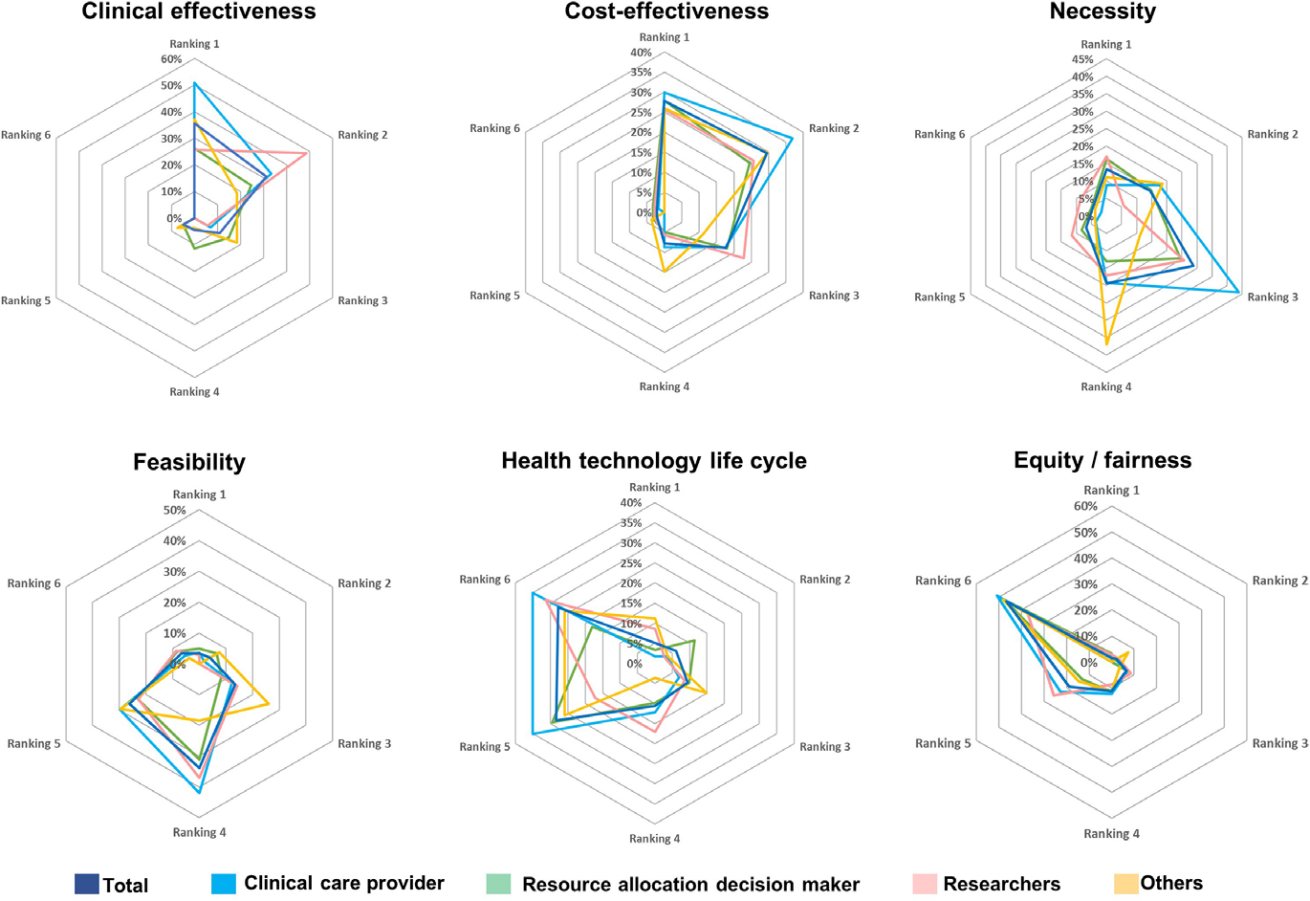
Criteria ranking in assessing disinvestment candidates.

In assessing candidates for disinvestment, respondents have ranked six criteria based on the options given in the survey (Figure 2). The top priority was evidence of clinical effectiveness, such as treatment effects and safety, changes in quality of life before and after intervention, and diagnostic accuracy. Evidence related to the program’s cost and cost-effectiveness, which included the operating expenses compared to its benefits and the maintenance costs of a specific health technology, ranked second in importance. Sub-group analysis revealed that researchers prioritized evidence on cost and cost-effectiveness above clinical effectiveness criteria, resulting in a higher ranking for the former. Following that were the necessity and feasibility of assessing the disinvestment candidates, which include the presence of an alternative to replace or displace the candidate, the availability of data for analysis, and support by patients or the public in discontinuing treatment. The fifth rank was the health technology life cycle, which looks at obsolete technologies, legacy items, and low uptake or utilization of therapies or interventions. The survey results revealed that despite their crucial role in healthcare decision-making, equity and fairness received the lowest priority rating for disinvestment criteria. However, this does not imply that the criteria are unimportant. In fact, when enquiring about the primary concern with the implementation of the disinvestment decision, several participants expressed worries about how disinvestment will affect treatment alternatives for vulnerable or disadvantaged groups in society.

### Perception on disinvestment initiative in Malaysia

**Figure 3.**
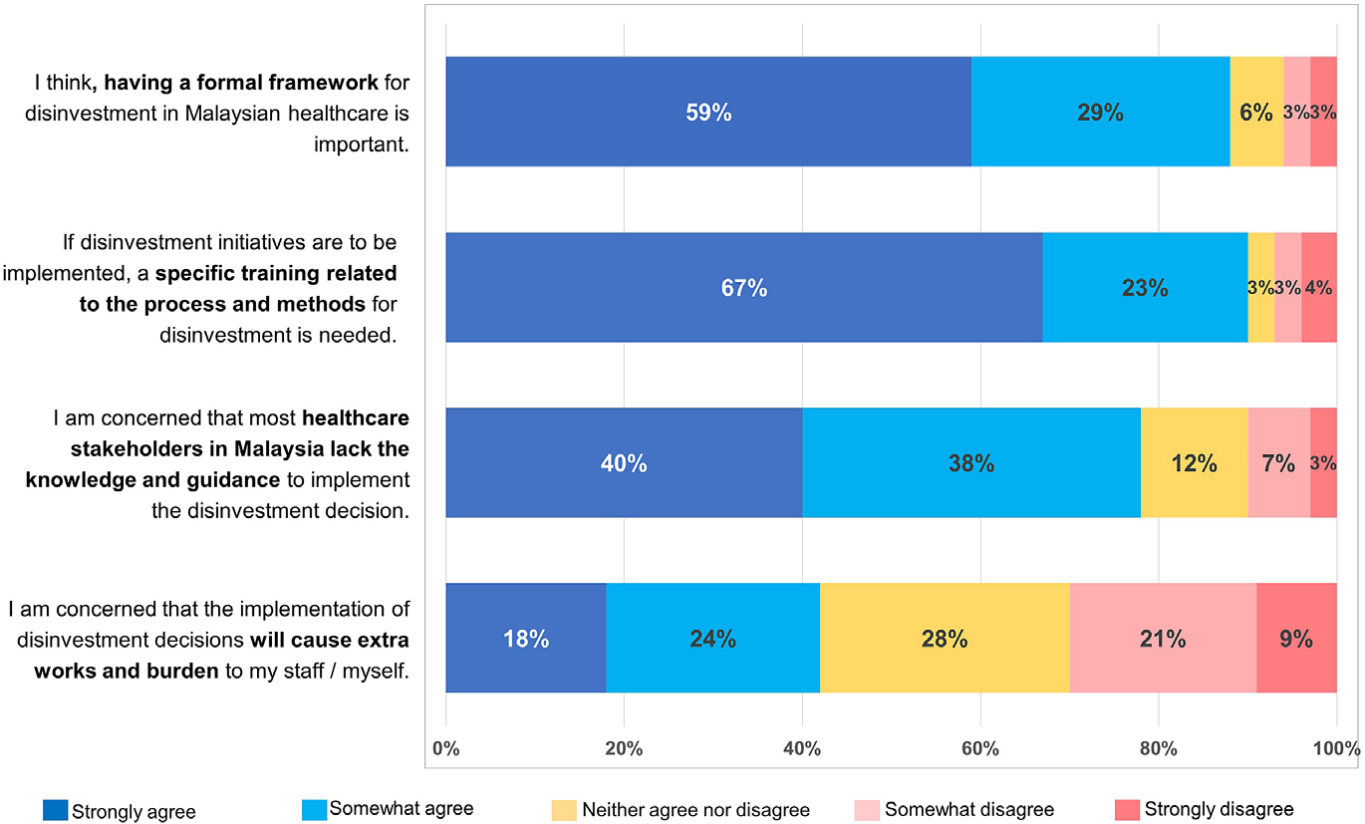
Perception on implementation of disinvestment initiatives in Malaysia.

In terms of acceptance and expectation, the majority (59 percent) of the respondents strongly agreed that there is a need for a formal framework for disinvestment *to evaluate and monitor previous decisions, to improve quality of care, and to implement a priority-based resource allocation process* (Figure 3 and Supplementary 10). Training is essential for the successful initiation of the disinvestment program, as indicated by 67 percent strongly agreeing. This aligns with the third component of insufficient knowledge among healthcare stakeholders on disinvestment, both in terms of performing assessments and implementing decisions. Respondents had varied reactions (agree, neither agree nor disagree, and disagree) when asked about the potential increase in workload due to disinvestment initiatives, indicating a reduced resistance to assuming the responsibility for implementing disinvestment decisions.

A sub-group analysis of stakeholders’ perspectives on implementing disinvestment initiatives in Malaysia was conducted based on respondents’ years of experience, and no difference was observed in the percentages of agreeing, neither agreeing nor disagreeing, and disagreeing on the four questions asked in the survey (see Supplementary 11).

Other responses to the stakeholders’ expectations on the implementation of the disinvestment framework in Malaysia are outlined in Supplementary 12, which includes the provision of training and awareness platforms for stakeholders, the development of a health policy for disinvestment, improvements in quality of care and resource allocation, as well as a transparent and comprehensive process of disinvestment.

### Facilitators and barriers in implementation of disinvestment process

Eighty-six percent of respondents identified organizational culture, particularly in terms of quality improvement and willingness to change, along with good leadership, as the key component in facilitating the disinvestment process, placing it as a top priority. The second facilitator is the involvement of key stakeholders responsible for healthcare decision-making, such as organization leaders, budget holders, clinical care providers, patients or their representatives, and the public. This would allow for a wider range of viewpoints to be considered, promoting ownership and acceptance of disinvestment decisions. The establishment of a transparent and robust methodology for disinvestment and the integration of the local context into the formulation of recommendations for disinvestment were identified as additional facilitators. A sub-group analysis based on stakeholder roles revealed slight differences in the order of facilitators in implementing disinvestment initiatives (Supplementary 13).

**Figure 4.**
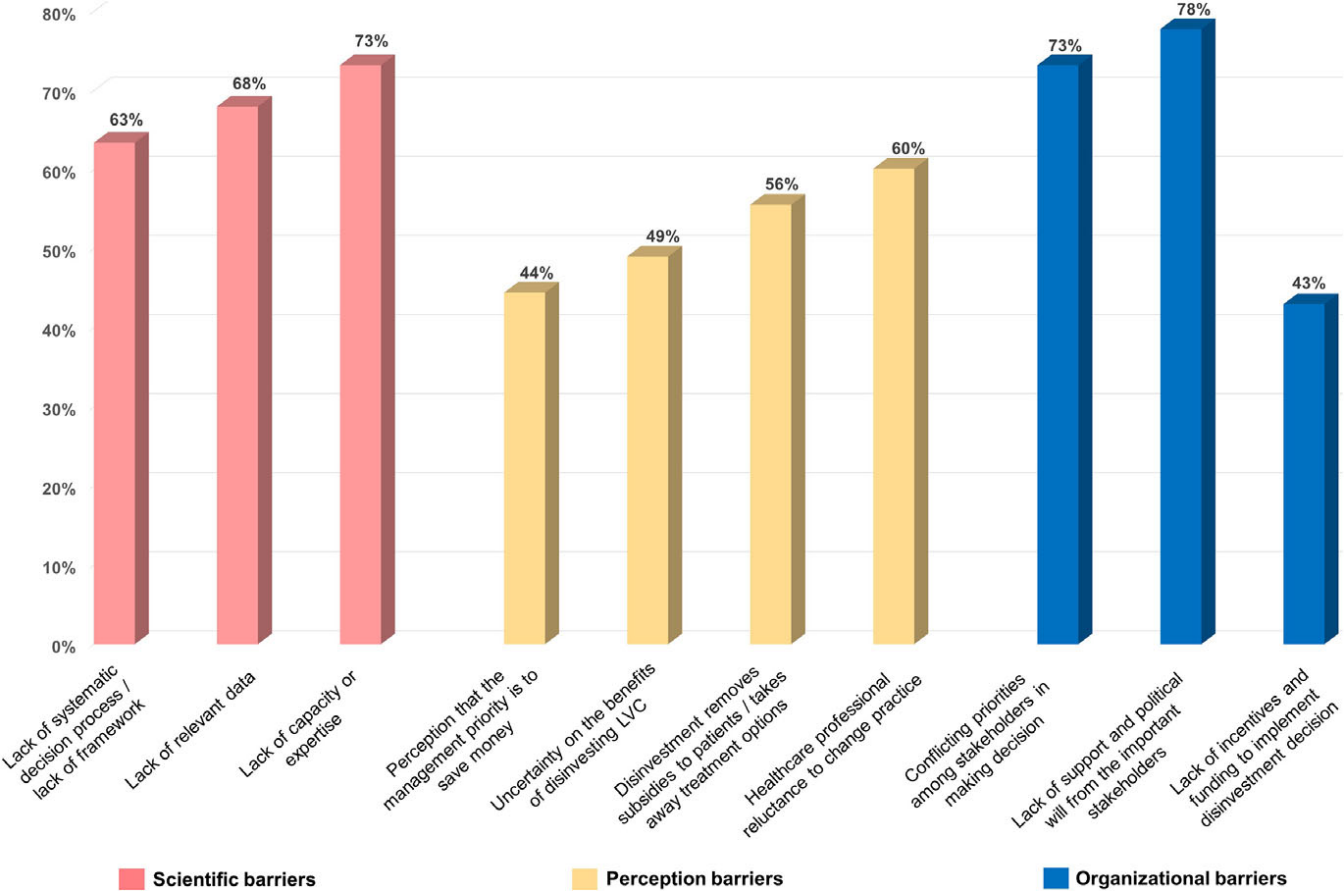
Barriers and challenges in implementation of disinvestment process.

The challenges in adopting the disinvestment process were categorized into scientific, organizational, and perceptual barriers (Figure 4). Seventy-eight percent of respondents identified the primary constraint as the lack of support and political will from stakeholders, followed by the high occurrence of conflicting priorities in decision-making and a lack of capacity or expertise in conducting the process (73 percent, respectively). Furthermore, 68 percent of respondents concurred that the absence of relevant data to support disinvestment decisions exacerbates the challenges.

## 4. Discussion

This online survey highlights the views of 153 key stakeholders in Malaysia regarding healthcare disinvestment. It focuses on their experiences and perceptions of implementing the program and aims to identify factors that can either support or hinder the acceptance of this initiative. By capturing diverse stakeholder perspectives in the early phase of planning, this research has generated valuable insights to inform future national implementation strategies, thereby fostering clarity and leading to a more robust and inclusive framework pertaining to disinvestment activities.

In making judgments to de-implement clinical practice, healthcare professionals rely on various factors, such as updated evidence, patient expectations and characteristics, economic and regulatory considerations, as well as their own expertise, clinical experience, and decorum (20). Engaging stakeholders early in the development of policy frameworks related to disinvestment in healthcare is crucial to ensuring that a diverse perspective is considered. This is the primary strength of our research, which is the first of its kind in relation to disinvestment initiatives in Malaysia and one of a few studies conducted in LMICs. We engaged healthcare stakeholders early in the process by gathering their perspectives from the planning phase of the proposed health policy. This is highly relevant at all levels of governance, although the implementation and method of assessment for disinvestment candidates could be different depending on the context and purpose of the activity.

In comparing the results of our survey with findings from LMICs, efforts have been made to identify similar studies from other countries on healthcare disinvestment and stakeholder perspectives on its implementation. Notably, only pertinent studies from Argentina, Mexico, and Brazil (21, 22) are available, providing information on the current state of disinvestment activities in these countries. Another paper described barriers and possible solutions in implementing the Choosing Wisely framework in LMICs with a mention of Tanzania’s experience (23). However, these articles did not incorporate the viewpoints of the key stakeholders. Other similar studies are mainly from high-income countries, particularly Canada and European region (14, 24-27).

To date, our understanding of priority setting and resource allocation (PSRA) in Malaysian healthcare has been restricted to the incorporation of criteria-based decision-making in HTA processes for funding and investment purposes, which have been established for more than two decades in the Malaysian public healthcare system (8, 9). The current study provides information on the criteria that respondents consider relevant to conducting evaluations regarding LVC de-implementation, which may influence decisions on disinvestment. Based on the priority of the criteria, we identified that clinical effectiveness and cost-effectiveness evidence are the two most important components, among others, which are also consistent with the criteria used in other PSRA frameworks (25) and the disinvestment processes in other countries (12, 28). This significant finding emphasizes the importance of these criteria in shaping methodological analyses and decision frameworks for healthcare disinvestment in Malaysia. By acknowledging these key components as paramount, we can develop a robust and equitable approach to the framework for efficient healthcare resource allocation while optimizing patient outcomes.

The majority of the respondents rated equity and fairness as the lowest among all other options. This finding is especially unexpected as disinvestment means reallocating resources from current services, which could worsen problems connected to access and delivery of health care, especially involving the elderly and patients with rare diseases or who are terminally ill. Disinvestment is a part of a larger ongoing initiative to improve healthcare for vulnerable patient groups by addressing gaps in care delivery, hence we still need to address important questions about how de-implementation and health equity intersect (29). Therefore, it is crucial to prioritize equity considerations in disinvestment discussions to ensure that decisions are inclusive of the well-being of all population segments, especially those most susceptible to adverse health impacts. By understanding these important attributes, policymakers can use this information to increase public support for disinvestment by strategically choosing suitable measures and effectively communicating disinvestment decisions.

Disinvestment in healthcare can be intricate and challenging due to multiple barriers that exist at different levels. Our research identified key barriers to implementing disinvestment initiatives, including organizational and scientific challenges related to support, political will, and conflicting priorities among stakeholders, as well as a lack of expertise, data, and a systematic framework for assessment. This is similar to findings from previous studies (24, 30, 31) which eventually hinder decision-makers from accepting and supporting disinvestment initiatives. Another possible barrier that is not captured in our study is the political and public perception, as disinvestment can be an emotive and contentious issue (32). Decision-makers may face pushback from the public, healthcare professionals, and special interest groups; making it difficult to implement and sustain disinvestment efforts. Therefore, integrating the local context into the formulation of recommendations for disinvestment is pivotal. Recognizing the unique healthcare landscape, cultural factors, and resource constraints of a particular region ensures that disinvestment strategies are tailored to address local needs and priorities effectively. By addressing these barriers and embracing the facilitators, it is possible to navigate the complexities of disinvestment with greater efficiency and acceptance from all stakeholders (33).

In terms of the acceptance and expectation of the disinvestment initiative, most respondents agree that there is a need for a defined, formal framework and guidelines for disinvestment. In this argument, we believe that healthcare professionals do not necessarily require guidance or instructions from others. They may be unwilling to confront the challenging issue, as defending it can lead to a complicated and messy situation. Establishing a clear framework for disinvestment enhances accountability and transparency in its implementation. There will always be individuals who oppose the disinvestment decision, and the guidelines will ultimately provide a level of protection beyond just counsel.

### Strengths and limitations of the survey

This survey encompassed all levels of governance and administration within the Malaysian public healthcare system, including national, regional, and state levels, academics, as well as individual facilities such as hospitals, primary clinics, and departmental levels. Moreover, budgetary issues and resource distribution often link to disinvestment discussions. Therefore, the most effective method is to engage with budget holders and key officials in the Ministry of Health. In our survey, snowball sampling is critical to include healthcare workers with decision-making experience in resource allocation, even if they are not in leadership roles like unit manager, director, or executive committee member. We also included professionals and practitioners who specialize in specific healthcare areas such as community care, pharmaceuticals, mental health, and clinical support services.

The importance of this study on healthcare disinvestment in Malaysia is demonstrated by the substantial number of survey responses and the inclusion of key stakeholders, given the rarity of this issue in the country. Despite the unfamiliarity of the topic, the responses are insightful. Participants emphasized the importance of promptly implementing disinvestment initiatives, especially within the constrained healthcare budget and resources. Reflectively, this research successfully attracted stakeholders’ attention due to its unique nature and the significant relevance of the topic, particularly in the post-COVID period, which had not been previously addressed. While we could not measure the response rate, we consider prioritizing an adequate number of representative respondents more crucial than achieving a high response rate, as recommended by a meta-analysis examining the overall response rate of online surveys in published research (19).

This study is constrained by the possibility of respondent bias. The main limitation of our study was associated with the methodology employed in our survey. The survey respondents were predominantly stakeholders in the public healthcare sector. Hence, it may be restricted to perceptions and activities within public health care facilities, while excluding the private sector. It is important to note that the assumptions and inputs in this research may not apply to the entire Malaysian population. Furthermore, there is limited awareness of healthcare disinvestment in Malaysia, leading to a significant percentage of survey participants withdrawing when questioned about their comprehension of the term “disinvestment in healthcare.” Hence, the findings of our study should be taken with caution, as there is still a risk of response bias due to the insufficient sample size resulting from the non-measured response rate. Our findings are limited to healthcare professionals and do not incorporate the perspectives of the public or patients in the country. However, previous studies have indicated that citizens were more supportive of accepting healthcare disinvestment compared to those who viewed it as less significant (34).

We also recognized the insufficient information on the small-scale disinvestment efforts in Malaysian healthcare systems. This survey did not offer a comprehensive understanding of the processes and methods used to assess the reported disinvestment activities. Further clarification on the appropriateness and adaptability of methodologies used would be beneficial for knowledge transfer, as would developing a policy-focused methodological analysis for disinvestment in Malaysia. Hence, we extended the research by conducting key informant interviews to explore further the components related to methodological analysis.

### Directions for future research

The outcomes from this research project may support the need for improving or enhancing existing tools used in disinvestment, such as program budgeting marginal analysis and HTA, or possibly offer another innovative method beyond these two processes. Additional information is needed to prevent fairness or equity from being compromised by a lack of awareness of the boundaries on implementing disinvestment in healthcare, which could be a potential research project in the future.

Another potential research area is on patient and public perspectives in healthcare disinvestment, which requires a specific study of its own due to the complexity of shared decision-making between care providers and patients. Therefore, we suggest future research to address patient and public viewpoints on the de-implementation of LVC and its societal impact.

## 5. Conclusions

In general, healthcare stakeholders in Malaysia perceived disinvestment as a process of withdrawing or reducing healthcare funding by reallocating resources from inefficient services and reinvesting in high-value technology. Small-scale and informal disinvestment activities were documented in the Malaysian health system at various levels of care, but an organized and structured approach is still lacking. The criteria for the disinvestment process should include evidence of clinical effectiveness and cost-effectiveness, the necessity and practicality of disinvesting the intervention from the system, the health technology life cycle, and equity or fairness. The majority concur that disinvestment requires a formal framework involving key stakeholders for guidance, and training on the method is crucial for its acceptance. Implementing disinvestment programs is challenging due to a lack of political will and organizational support, conflicting stakeholder agendas, and a lack of skills and relevant data to evaluate candidates for disinvestment. Future research should link methodological analysis to healthcare disinvestment as part of the resource allocation strategy and investigate approaches to incorporating equity and fairness in assessing disinvestment candidates.

## Supporting information

Kamaruzaman et al. supplementary material Kamaruzaman et al. supplementary material

## Supplementary material

The supplementary material for this article can be found at http://doi.org/10.1017/S0266462324004665.
