# Supplementary material for: Stakeholders’ perspectives on disinvestment of low-value healthcare interventions and practices in Malaysia: an online survey
Source: Int J Technol Assess Health Care. 2024 Nov 15;40(1):e57. doi: 10.1017/S0266462324004665 (PMC11579699; doi:10.1017/S0266462324004665)
Supplement: Kamaruzaman et al. supplementary material [file S0266462324004665sup001.pdf]

SUPPLEMENTARY DOCUMENTS

Stakeholders’ perspectives on disinvestment of low-value healthcare interventions and practices in Malaysia: An online survey

Supplementary 1: Examples of comments from face validity of survey questionnaire

Panel	Comments
FV1	• Time taken: 30 minutes<br>• I think your participant information sheet is a very nice idea because without them, it’s difficult to understand the terms too.<br>• Especially Q11, 12, it took a while.<br>• I think for your target group interviewers, they might take longer time than me.<br>• Because they will fill more information (I did not fill the questions about Malaysian healthcare)<br>• Probably from 40 minutes and up to one hour to finish it.<br>• From my experience, if stakeholders get this form, they will use it as a chance to ‘complaint’ and write a lot.
FV2	• Time taken: 22 minutes<br>• It is generally clear and easy to use but I have some specific comments below:<br>• Q12 - maybe add a bit more to explain what you mean here - priority when you are assessing or when you are triggering disinvestment?<br>• Q14 - do you only want people to answer this if they have experience of disinvestment?<br>• Q14 - not allowing me to put numbers in - just crosses<br>• Q16 - is this more complicated than it needs to be? I was wondering about the relevance of the red cell & serum folate<br>• Q17 - 20 - maybe set out how you want people to indicate which answer they want - highlight?<br>• Q18 - think this should be the agree/disagree answers<br>• Q21 - do you need this question to be more than one question - should a disinvestment in another country automatically trigger a process in Malaysia? What should the process look like? Use the language around adaptability as for HTA reports.

Supplementary 2: Examples of content validity index assessment of survey questionnaire

Question	Are the items representative of concepts related to disinvestment?				Are the items relevant to the concepts related to disinvestment?				Are the items clear in term of wordings? (clarity)				Comments *(Panel A, Program manager in MOH Malaysia)*
	1	2	3	4	1	2	3	4	1	2	3	4	
Q8	☐	☐	☐	☒	☐	☐	☐	☒	☐	☐	☒	☐	Clear question and a good start for the domain.
Q9	☐	☒	☐	☐	☐	☐	☒	☐	☐	☐	☒	☐	Suggest having more options for critical successful factor to balance the factors related to unobtainable target.
Q10	☐	☐	☐	☒	☐	☐	☐	☒	☐	☐	☐	☒	Interesting question, may trigger the respondents to write a short essay on their opinion.
Q11	☐	☐	☐	☒	☐	☐	☐	☒	☐	☐	☒	☐	Very relatable. “One-in-one-out” policy might be confusing/unfamiliar for some respondent.
Q12	☐	☐	☐	☒	☐	☐	☐	☒	☐	☐	☐	☒	The explanations given in bracket are helpful.
Q13	☐	☐	☒	☐	☐	☐	☐	☒	☐	☐	☒	☐	Suggest separating “decision makers/key leaders” with “budget holders/resource managers”
Q14	☐	☐	☐	☒	☐	☐	☐	☒	☐	☐	☐	☒	Consistency in ranking must be decided.
Q15	☐	☐	☐	☒	☐	☐	☐	☒	☐	☐	☐	☒	Same as Q14, consistency in ranking.
Q16	☐	☐	☒	☐	☐	☐	☒	☐	☐	☒	☐	☐	Vignette too long. Maybe difficult to understand for non-clinical personnel. Suggest to simplify the text.
Q17	☐	☐	☒	☐	☐	☐	☐	☒	☐	☐	☐	☒	Instruction should be included.
Q18	☐	☐	☒	☐	☐	☐	☐	☒	☐	☐	☒	☐	For Q17-Q20, suggest having 5 scale, including neutral/neither agree nor disagree.
Q19	☐	☐	☐	☒	☐	☐	☐	☒	☐	☐	☐	☒	Same as above.
Q20	☐	☐	☐	☒	☐	☐	☒	☐	☐	☐	☐	☒	I expect majority will answer agree/strongly agree
Q21	☐	☐	☒	☐	☐	☐	☐	☒	☐	☐	☐	☒	Looks like prompt question. Suggest making it open to allow respondents give their opinion.

Ratings:
1 = not relevant/not clear
2 = item need revision
3 = relevant but need minor revision/clear but need minor revision
4 = very relevant/very clear or unambiguous

Adapted from:

1. *Polit DF, Beck CT. The content validity index: are you sure you know what's being reported? Critique and recommendations. Research in nursing & health. 2006 Oct;29(5):489-97.*
2. *Kumar PR, Yee A, Francis B, Danaee M. Adaptation and Validation of a scale to Assess Knowledge, Attitudes, and Perceptions of Healthcare Workers Towards Alcohol Withdrawal and Its Detection. International Journal of Mental Health and Addiction. 2022 Oct;20(5):3006-21.*

Supplementary 3: Overall item content validation index (I-CVI) and Kappa statistic for survey questionnaire (representativeness, relevancy and clarity)

Question	Panel 1	Panel 2	Panel 3	Panel 4	Panel 5	Panel 6	Total Agreement (A)	Total Expert (N)	I-CVI	Kappa	Results
**Q8**	Y	Y	Y	Y	Y	Y	6	6	**1.000**	**1.000**	**Validated**
**Q9**	N	Y	Y	Y	Y	Y	5	6	**0.833**	**0.816**	**Validated**
**Q10**	Y	Y	Y	Y	Y	Y	6	6	**1.000**	**1.000**	**Validated**
**Q11**	Y	Y	Y	Y	Y	Y	6	6	**1.000**	**1.000**	**Validated**
**Q12**	Y	Y	Y	Y	Y	Y	6	6	**1.000**	**1.000**	**Validated**
**Q13**	Y	Y	N	Y	Y	Y	5	6	**0.833**	**0.816**	**Validated**
**Q14**	Y	Y	Y	Y	Y	Y	6	6	**1.000**	**1.000**	**Validated**
**Q15**	Y	Y	Y	Y	Y	Y	6	6	**1.000**	**1.000**	**Validated**
**Q16***	N	N	Y	N	Y	Y	3	6	**0.500**	**0.273**	**Corrected (clarity)**
**Q17**	Y	Y	Y	N	Y	Y	5	6	**0.833**	**0.816**	**Validated**
**Q18**	Y	Y	Y	N	Y	Y	5	6	**0.833**	**0.816**	**Validated**
**Q19**	Y	Y	Y	N	Y	Y	5	6	**0.833**	**0.816**	**Validated**
**Q20**	Y	Y	Y	N	Y	Y	5	6	**0.833**	**0.816**	**Validated**
**Q21**	Y	Y	Y	Y	Y	Y	6	6	**1.000**	**1.000**	**Validated**
**Proportion relevant**	**0.857**	**0.929**	**0.929**	**0.643**	**1**	**1**					

Item content validity index (I-CVI): > 0.79 (validated), 0.70–0.79 (needs revision), < 0.70 (eliminated).
Kappa (k): excellent (≥0.74), good (0.60 to 0.73), moderate (0.40 to 0.59), poor (≤0.39).
*Question 16 (clinical vignette) was revised to ensure clarity of the case example and validated with the panel after the amendment.

Supplementary 4: Final survey questionnaire

Stakeholder perspectives on disinvestment of low-value healthcare interventions and practices in Malaysia: A survey of healthcare key informants

The participants will have to complete this online survey which includes a series of open and closed questions, and comprises of **5 sections**:

A. Background information
B. Knowledge and perceptions on disinvestment in healthcare
C. Disinvestment initiatives within organisation / workplace
D. Facilitators and challenges in implementation of disinvestment activity and programme
E. Receptivity and expectation on implementation of disinvestment initiatives within Malaysian health care system

By clicking the button below, you acknowledge:

- Your participation in the study is voluntary
- All data and information that you provide will be kept confidential and will only be used for the purpose of this research project
- You are aware that you are free to withdraw at any time for any reason

Do you agree and consent to take part in this survey?

☐ Yes, I consent to begin the study.

☐ No, I do not consent and I disagree to participate. *(exit from survey)*

Section A: Background Information

Q.1 Your current workplace:

____

Q.2 What is your primary professional role within the health care? *(Choose all that apply)*

☐ **Resource allocation decision makers / Budget holders**
(e.g. Programme Managers, Hospital Directors, Head of Clinical Services, others)
Please specify: ______________________________________

☐ **Clinical care providers** *(e.g. clinician, physician, pharmacist, nurse, allied health)*
Please specify: ______________________________________

☐ **Researchers / academia / experts in Health Technology Assessment (HTA) and/or Health Economics and/or Health Services** *(panels of Technical Advisory Committee, university lecturer, interested research groups working in HTA, health economics or health administration/ management)*

Please specify: ____________________

☐ **Other than mentioned above**

Please specify: ____________________

Q.3 Experience in the above role (years):

____

Q.4 Level of governance / decision-making in health care system you are most familiar with: *(Choose all that apply)* *[Note: you do not need to be directly involved in decision-making]*

☐ National level

☐ State / Federal territory level

☐ Regional level (e.g., health authority, health district, health region)

☐ Single organisational level (e.g., hospital / institute, primary care, community organisation, residential care facility)

☐ Other than mentioned above

Please specify: ____________________

Q.5 What are the types of health technologies or scope in the context of decision-making that you are familiar with? *(Choose all that apply)*

☐ Pharmaceuticals / drugs / medicines

☐ Non-pharmaceuticals (e.g., medical devices, digital technologies, medical procedures, screening programmes, diagnostic devices, health programmes)

Please specify: ____________________

☐ Specific fields of care (e.g., primary care, cancer, public health).

Please specify: ____________________

☐ Work force / human resources

☐ Other than mentioned above

Please specify: ____________________

Section B: Knowledge and perceptions on disinvestment in healthcare

Q.6 What do you understand by the term “disinvestment in healthcare”?

Q.7 Do you think formal disinvestment process is needed in healthcare, related to the context of your level of governance?

☐ **Yes** (please state your reason)

☐ **No** (please state your reason)

Q.8 From your understanding, the purpose of disinvestment includes: *(Choose all that apply)*

☐ Cost-saving to health care system by removing unnecessary spending

☐ Reduction of the waste of resources by minimising ineffective spending

☐ Reinvestment in health technologies or interventions which have higher values

☐ Shifting resource from one area of healthcare to another (reallocation of resource)

☐ Removing “no- or low-value” technologies / treatments from clinical practice

☐ Improving quality of care and widening service provision

☐ Increase benefits to patients and community as a whole

☐ Ensuring optimum clinical effectiveness and safe treatments provided to patients

☐ Informed decision-making in addressing budgetary gaps or limited resources

☐ Reducing variation in clinical practice by limiting health technologies / treatments / practices in the benefits package / guidelines

☐ Other than mentioned above
Please specify: ____

Section C: Disinvestment initiatives within organisation / workplace

Q.9 Do you have any previous experience with disinvestment / resource reallocation activity in your organisation/institution/department?

☐ **Yes** (please provide example) *(go to Q.9a)*

☐ **No** *(go to Q.10)*

Q.9a *(If Yes in Q.9)* Does it achieve the purpose and goal of this activity within your organisation / institution / department?

☐ **Yes** *(go to Q.9b)*

☐ **No** *(go to Q.9c)*

Q.9b *(If Yes in Q.9a)* What are the factors that contribute to the success of the disinvestment activity? *(Choose all that apply)*

☐ Participation of a diverse range of stakeholders with varying roles and expertise

☐ Use of systematic and acceptable method or process for decision-making in disinvestment

☐ Availability of evidence and local data to support decision-making in disinvestment

☐ Presence of strong leadership (including funding) to ensure implementation of disinvestment decision taking place

☐ Ongoing interactions, training and knowledge exchange among stakeholders to support implementation of disinvestment recommendations

☐ Other than mentioned above
Please specify: ________________________________

Q.9c *(If No in Q.9a)* What are the factors that contribute to the unobtainable target of this activity? *(Choose all that apply)*

☐ Reluctance from stakeholders (e.g. clinician, care provider) to change practice

☐ Lack of funding for implementation of disinvestment activity or decision

☐ Lack of training on how to conduct the assessment for disinvestment purpose

☐ Lack of support from key leader(s) to implement disinvestment decision

☐ Relevant data are not available to demonstrate inefficiency of a health technology or evidence of low-value care for the purpose of disinvestment

☐ Other than mentioned above
Please specify: ____

Q.10 Do you think disinvestment or reassessment of health practices and technologies should be carried out on a regular basis as part of the organisational / departmental activities? If so, why? *[Note: you may answer even if you have no experience in disinvestment]*

☐ **Yes** (please state your reason)

☐ **Maybe** (please state your reason)

☐ **No** (please state your reason)

Q.11 From your opinion or previous experience, what are the **triggers in initiating assessment for disinvestment activity**? *(Choose all that apply)*

☐ Change in budgetary planning / resource allocation

☐ Presence of new evidence on effectiveness or safety on a health technology

☐ Variation in practice at care level *(e.g. conflicting with clinical practice guidelines)*

☐ Evidence of public interest or controversies *(e.g. equity issues)*

☐ Evidence of harmful effect to patients or safety issues

☐ Decreased frequency of use / prescription / utilisation of a health technology

☐ Presence of new technology to be included in the service hence the low-value technology need to be taken out *("one-in-one-out" policy)*

☐ Other than mentioned above *(please specify)*

____

Q.12 What criteria should be considered as **priorities** in conducting assessment for disinvestment? Please rank the statements with rank 1 is the highest priority.

☐ **Evidence of clinical effectiveness** *(effect and safety of treatment, quality of life before and after treatment, necessity for further research, clinical practice, patient relevance)*

☐ **Evidence of cost-effectiveness** *(high cost but low benefits / outcomes, cost for maintenance or implementation higher than expected benefits)*

☐ **Necessity** *(burden of illness, medical necessity, no alternative treatment, individual responsibility / lifestyle)*

☐ **Feasibility** *(support by society in discontinuing treatment, presence of alternative, availability of mechanism / data to support reassessment)*

☐ **Health technology life cycle** *(legacy items or interventions that had never been assessed before, obsolete technologies, approved to be used for research purpose but low uptake by patients)*

☐ **Equity / fairness** *(treatment affects a certain group of people in society such as vulnerable group or rare disease; end-of-life care, treatment for life-threatening condition in young people)*

☐ **Other than mentioned above** *(please specify)*

____

Q.13 Who should be the stakeholders involved in disinvestment activity or resource reallocation? *(Choose all that apply)*

☐ Decision-makers / key leaders

☐ Budget holder / funding managers

☐ Clinical care providers (clinicians or physicians, pharmacists, nurses, allied health)

☐ Public society / community

☐ Patient or patient's representative (e.g. support group, cancer patient society)

☐ Other than mentioned above *(please specify)*

____

Section D: Facilitators and challenges in implementation of disinvestment activity

Q.14 In your opinion, what are the facilitators for the implementation of processes for disinvestment or resource reallocation of low-value care that you can identify in the context you are involved with? Please rank the statements with rank 1 is the highest priority.

- ☐ Involvement of various stakeholders in healthcare
- ☐ Organisational culture for improvement in quality of care and openness to change, including strong leadership.
- ☐ Transparent and robust method for identification, prioritisation, and assessment of candidates for disinvestment
- ☐ Integrating local context in formulating recommendation for disinvestment purpose
- ☐ Other than mentioned above *(please specify)*

  ____

Q.15 In your opinion, what are the barriers or challenges for the implementation of processes for disinvestment or resource reallocation of low-value care that you can identify in the context you are involved with? Please choose maximum of 7 options.

- ☐ Lack of expertise to assess a health technology / practice / medicine for disinvestment decision
- ☐ Lack of support from the important stakeholders (e.g. refusal from care provider to remove certain practices / technologies / legacy drugs)
- ☐ Perceptions that disinvestment removes subsidies to patient or ‘takes away’ treatment options from patient.
- ☐ Lack of relevant data to conduct assessment for disinvestment
- ☐ Conflicting priorities among stakeholders in making decision
- ☐ Uncertainty over the benefits of the decision to disinvest low-value care
- ☐ Lack of systematic decision process for disinvestment (no available framework)
- ☐ Clinician reluctance to remove practices, thinking that disinvestment limits health providers' clinical autonomy and reduces prescriber treatment options
- ☐ Perception that the management priority is only to save money
- ☐ Lack of incentives and funding to implement disinvestment decision

Q.16 Please read the case scenario below and answer the following question:

As part of Quality Improvement initiatives in the Ministry of Health and in line with the call for implementation of value-based decision-making for resource allocation, Hospital X is encouraged to identify areas for improvement, where potential de-implementation of low-value practices (due to inefficiency or minimal benefit) can be suggested.

Concurrently, there is substantial rise in the test for vitamin B12 level in Hospital X. For a period of 6 months in 2022, the number of tests ordered for serum B12 reached 280 tests, doubled from the number of tests performed in 2021. This was alerted to the Head of Pathology and upon further investigation, it was found that most of the tests were done with the indication of “new episode of unexplained fatigue” among hospitalised adult patients. About 80% of tests were performed for the purpose of screening / diagnosing rather than monitoring. It was considered as unnecessary test and usually, patients with unexplained fatigue are treated symptomatically with oral B12 supplement without prior diagnostic test.

As the test for serum B12 was carried out together with red cell folate and serum folate, it incurred a higher cost compared to previous years. Hence, it is suggested that serum cobalamin test for “unexplained fatigue” should be considered for delisting from subsidised pathology test under public fund.

In your opinion, what are the factors that may influence your decision whether to disinvest or not in this scenario? Please rank the statements with rank 1 is the highest priority.

- ☐ Availability of evidence to support the assessment and decision
- ☐ Having something else to offer in compensating the de-listing of this practice (e.g. treatment with oral B12 without testing)
- ☐ Cost of current practice and cost of alternative strategy that will be implemented
- ☐ Patient factors and risk of not performing the test (e.g. prescribing B12 supplement without doing the test may be considered as over-treating)
- ☐ Feasibility to change practice, especially among the clinicians
- ☐ Other than mentioned above *(please specify)*

  ____

Section E: Receptivity and expectation of disinvestment initiatives within the Malaysian health care system

Please select one answer for each statement on your opinion regarding implementation of disinvestment initiatives within the Malaysian healthcare system:

Q.17 I think, having a formal framework for disinvestment in the Malaysian health care system is important.

1	2	3	4	5
Strongly disagree	Somewhat disagree	Neither agree nor disagree	Somewhat agree	Strongly agree

Q.18 If disinvestment initiatives are to be implemented, a specific training related to the process and methods for disinvestment is needed.

1	2	3	4	5
Strongly disagree	Somewhat disagree	Neither agree nor disagree	Somewhat agree	Strongly agree

Q.19 I am concerned that most healthcare stakeholders in Malaysia lack the knowledge and guidance to implement the disinvestment decision.

1	2	3	4	5
Strongly disagree	Somewhat disagree	Neither agree nor disagree	Somewhat agree	Strongly agree

Q.20 I am concerned that the implementation of disinvestment decisions will cause extra works and burden to my staff / myself.

1	2	3	4	5
Strongly disagree	Somewhat disagree	Neither agree nor disagree	Somewhat agree	Strongly agree

Q.21 If a health technology has been disinvested in other setting (e.g. country or region), should the same technology be reassessed locally for disinvestment? Why?

☐ **Yes** (please state your reason)

☐ **No** (please state your reason)

Q.22 If formal process or framework for disinvestment of health technologies or reassessment of low-value care is to be developed by Ministry of Health Malaysia, what is your expectation from the process?

Q.23 If you have any other comments in relation to disinvestment in Malaysian healthcare system, please write them below.

Q.24 Do you know other healthcare professionals, decision-makers or researchers in Malaysia who may also be able to contribute to this project? Could you please list below their names and institutional affiliation (and/or email address)? Otherwise, please feel free to share the survey link with these potential participants.

Thank you for taking the time to complete this survey. We greatly value and appreciate your participation. For the researchers to gain a more in-depth perspectives on disinvestment initiatives and the process for its implementation in Malaysian healthcare, we wish to conduct **a semi-structured follow-up interview** (around 30-45 minutes) with healthcare stakeholders in Malaysia. If you are happy to participate in such an interview, please check the appropriate box below and leave your name and e-mail address. We will contact you for further information on the follow up interview.

☐	**Yes, I would like to participate in a follow-up interview (please give your name and email address / contact number)**
☐	**No, thank you.**

Supplementary 5: Checklist for Reporting of Survey Studies (CROSS)

Section/topic	Item	Item description	Reported on page #
**Title and abstract**			
Title and abstract	1a	State the word "survey" along with a commonly used term in title or abstract to introduce the study's design.	1
	1b	Provide an informative summary in the abstract, covering background, objectives, methods, findings/results, interpretation/discussion, and conclusions.	1
**Introduction**			
Background	2	Provide a background about the rationale of study, what has been previously done, and why this survey is needed.	1 & 2
Purpose/aim	3	Identify specific purposes, aims, goals, or objectives of the study.	2
**Methods**			
Study design	4	Specify the study design in the methods section with a commonly used term (e.g., cross-sectional or longitudinal).	2
Data collection methods	5a	Describe the questionnaire (e.g., number of sections, number of questions, number and names of instruments used).	2
	5b	Describe all questionnaire instruments that were used in the survey to measure particular concepts. Report target population, reported validity and reliability information, scoring/classification procedure, and reference links (if any).	2 and Supplementary 1-3
	5c	Provide information on pretesting of the questionnaire, if performed (in the article or in an online supplement). Report the method of pretesting, number of times questionnaire was pre-tested, number and demographics of participants used for pretesting, and the level of similarity of demographics between pre-testing participants and sample population.	2
	5d	Questionnaire if possible, should be fully provided (in the article, or as appendices or as an online supplement).	Supplementary 4
Sample characteristics	6a	Describe the study population (i.e., background, locations, eligibility criteria for participant inclusion in survey).	2
	6b	Describe the sampling techniques used (e.g., single stage or multistage sampling, simple random sampling, stratified sampling, cluster sampling, convenience sampling). Specify the locations of sample participants whenever clustered sampling was applied.	2
	6c	Provide information on sample size, along with details of sample size calculation.	2
	6d	Describe how representative the sample is of the study population (or target population if possible), particularly for population-based surveys.	2

Survey administration	7a	Provide information on modes of questionnaire administration, including the type and number of contacts, the location where the survey was conducted (e.g., outpatient room or by use of online tools, such as SurveyMonkey).	**2**
	7b	Provide information of survey's time frame, such as periods of recruitment, exposure, and follow-up days.	**2**
	7c	Provide information on the entry process:<br>–>For non-web-based surveys, provide approaches to minimize human error in data entry.<br>–>For web-based surveys, provide approaches to prevent "multiple participation" of participants.	**2**
Study preparation	8	Describe any preparation process before conducting the survey (e.g., interviewers' training process, advertising the survey).	**2**
Ethical considerations	9a	Provide information on ethical approval for the survey if obtained, including informed consent, institutional review board [IRB] approval, Helsinki declaration, and good clinical practice [GCP] declaration (as appropriate).	**3**
	9b	Provide information about survey anonymity and confidentiality and describe what mechanisms were used to protect unauthorized access.	**2 & 3**
Statistical analysis	10a	Describe statistical methods and analytical approach. Report the statistical software that was used for data analysis.	**3**
	10b	Report any modification of variables used in the analysis, along with reference (if available).	**NA**
	10c	Report details about how missing data was handled. Include rate of missing items, missing data mechanism (i.e., missing completely at random [MCAR], missing at random [MAR] or missing not at random [MNAR]) and methods used to deal with missing data (e.g., multiple imputation).	**NA**
	10d	State how non-response error was addressed.	**NA**
	10e	For longitudinal surveys, state how loss to follow-up was addressed.	**NA**
	10f	Indicate whether any methods such as weighting of items or propensity scores have been used to adjust for non-representativeness of the sample.	**NA**
	10g	Describe any sensitivity analysis conducted.	**3 (cross-tabulation, sub-group analysis)**
**Results**			
Respondent characteristics	11a	Report numbers of individuals at each stage of the study. Consider using a flow diagram, if possible.	**3, Table 1 & Supplementary 6**
	11b	Provide reasons for non-participation at each stage, if possible.	**3, Table 1 & Supplementary 6**

	11c	Report response rate, present the definition of response rate or the formula used to calculate response rate.	**NA**
	11d	Provide information to define how unique visitors are determined. Report number of unique visitors along with relevant proportions (e.g., view proportion, participation proportion, completion proportion).	**3, Table 1 & Supplementary 6**
Descriptive results	12	Provide characteristics of study participants, as well as information on potential confounders and assessed outcomes.	**3, Table 1**
Main findings	13a	Give unadjusted estimates and, if applicable, confounder-adjusted estimates along with 95% confidence intervals and p-values.	**NA**
	13b	For multivariable analysis, provide information on the model building process, model fit statistics, and model assumptions (as appropriate).	**NA**
	13c	Provide details about any sensitivity analysis performed. If there are considerable amount of missing data, report sensitivity analyses comparing the results of complete cases with that of the imputed dataset (if possible).	**NA**
**Discussion**			
Limitations	14	Discuss the limitations of the study, considering sources of potential biases and imprecisions, such as non-representativeness of sample, study design, important uncontrolled confounders.	**7**
Interpretations	15	Give a cautious overall interpretation of results, based on potential biases and imprecisions and suggest areas for future research.	**8**
Generalizability	16	Discuss the external validity of the results.	**7 & 8**
**Other sections**			
Role of funding source	17	State whether any funding organization has had any roles in the survey's design, implementation, and analysis.	**8**
Conflict of interest	18	Declare any potential conflict of interest.	**8**
Acknowledgements	19	Provide names of organizations/persons that are acknowledged along with their contribution to the research.	**8**

Supplementary Figure 6: Flowchart of survey invitation and responses

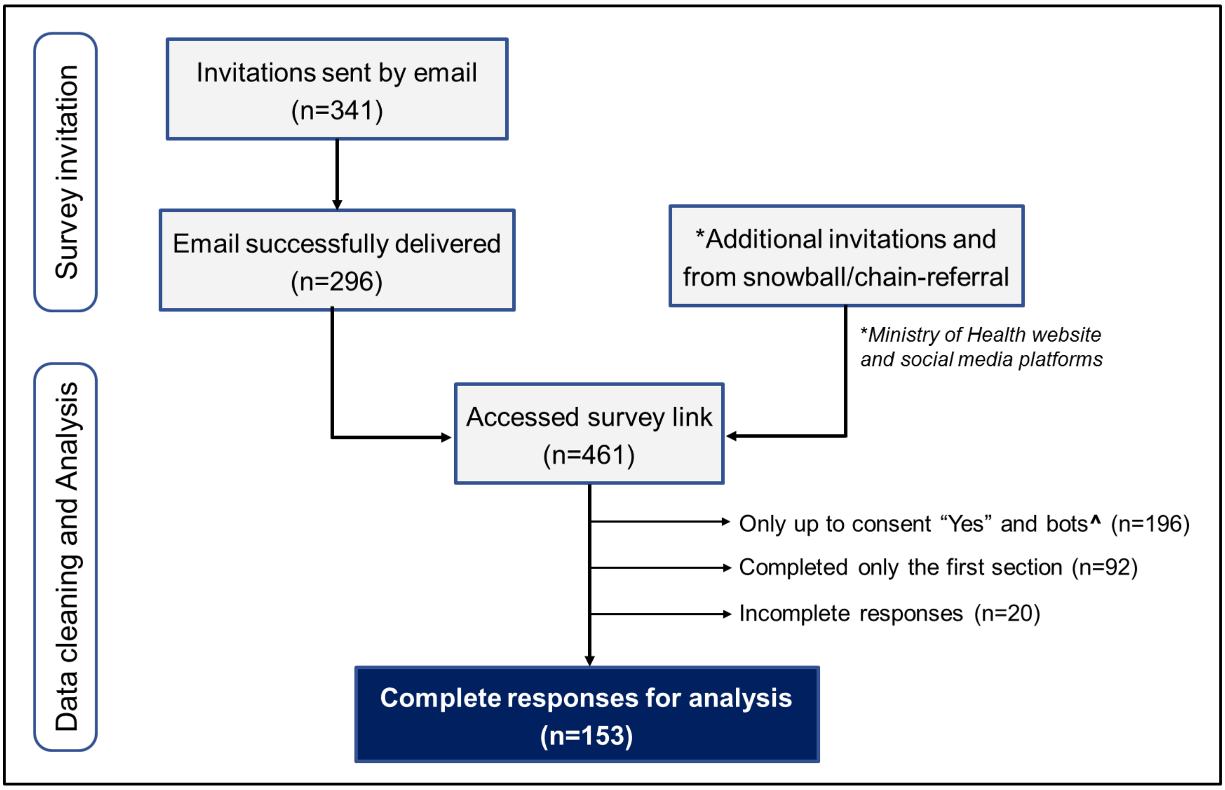

^Notes:

In our analysis, two indicators were used to identify whether the responses originated from bots or from human:

i. **Super-fast responses** (less than 3 minutes). For the completed survey, we checked the time to complete the survey one by one. The fastest time taken by the respondents to complete the survey was 9 minutes.

ii. **Duplicate IP addresses**. Bots often use the same IP address to answer surveys repeatedly in a short period of time.

'Bot detection function' was enabled in Qualtrics which allows us to track which responses are likely to be bots.

Supplementary 7: Disinvestment activity experiences by survey respondents

(sub-grouping using content analysis based on similarity of activities)

**A. Pharmaceuticals**	
1.	Removing non-essential drugs that were not used much / has no clinical evidence and cost-effectiveness evidence from health clinics and hospitals formulary lists
2.	Re-proportionate the amount of generic and original brand of medications for patients
3.	Streamlined the use of angiotensin receptor blockers (ARB) as there were too many different strengths and types in the formulary (de-listing, revise indication for some ARBs)
4.	Cutting budget for some basic medications in district health clinic
5.	Reassessment and Selection Strategy of Dipeptidyl Peptidase-4 Inhibitors for Ministry of Health Malaysia Medicine Formulary
6.	Re-assessment of inhalers for COPD and asthma for listing and de-listing from formulary
7.	Reducing the purchase of unnecessary medicine such as oral supplement
**B. Hospital management**	
8.	Outsourcing kitchen service to third party for patient meals preparation
9.	Re-zoning the laboratories in hospital
**C. Clinical and surgical procedures**	
10.	Re-evaluate the need for ‘routine’ blood & radiological investigations before surgery
11.	Laser treatment for hemorrhoidal disease (beneficial in early disease but too costly)
12.	Shifting from thrombolysis care to primary PCI for acute myocardial infarction
13.	Removing annual ECG checking for stable hypertensive patients during clinic appointment
**D. Medical devices and digital health**	
14.	Re-assessment of outdated medical appliances and systems (ventilators, ultrasound machine, critical care monitoring system) to replace with the latest machines and system
15.	Change of conventional x-ray to digital x-ray in primary care clinics
16.	Re-location of laser machine to specialised center
17.	Relocation of biochemical machine to other district hospital
**E. Community health and primary care**	
18.	Closure of 1Malaysia clinics, rebranding to community health clinics
19.	Disinvest some of the services in PeKa B40 programs which involve private GP
**F. Public health programmes**	
20.	Removal of Hepatitis B screening from Occupational and Safety Health activities / stop screening staff that was born after 1989
21.	Shift from pap smear to HPV DNA testing for cervical cancer screening
22.	Re-assessment of vaccinations programs for national policy
**G. Human resource and work force**	
23.	Reallocation of staff to community-based wellness hub
24.	Combining units / departments due to lack of staff and redundant job scope
**H. Others**	
25.	Re-assessment of health education tools and services available
26.	Re-evaluation of budget for staff training that provide minimal output, low benefits
27.	Shifting from manual / paper-based patient satisfaction survey to online platform / QR code

Supplementary Figure 8: Complexity in decision-making related to reported disinvestment activities in Malaysian healthcare system

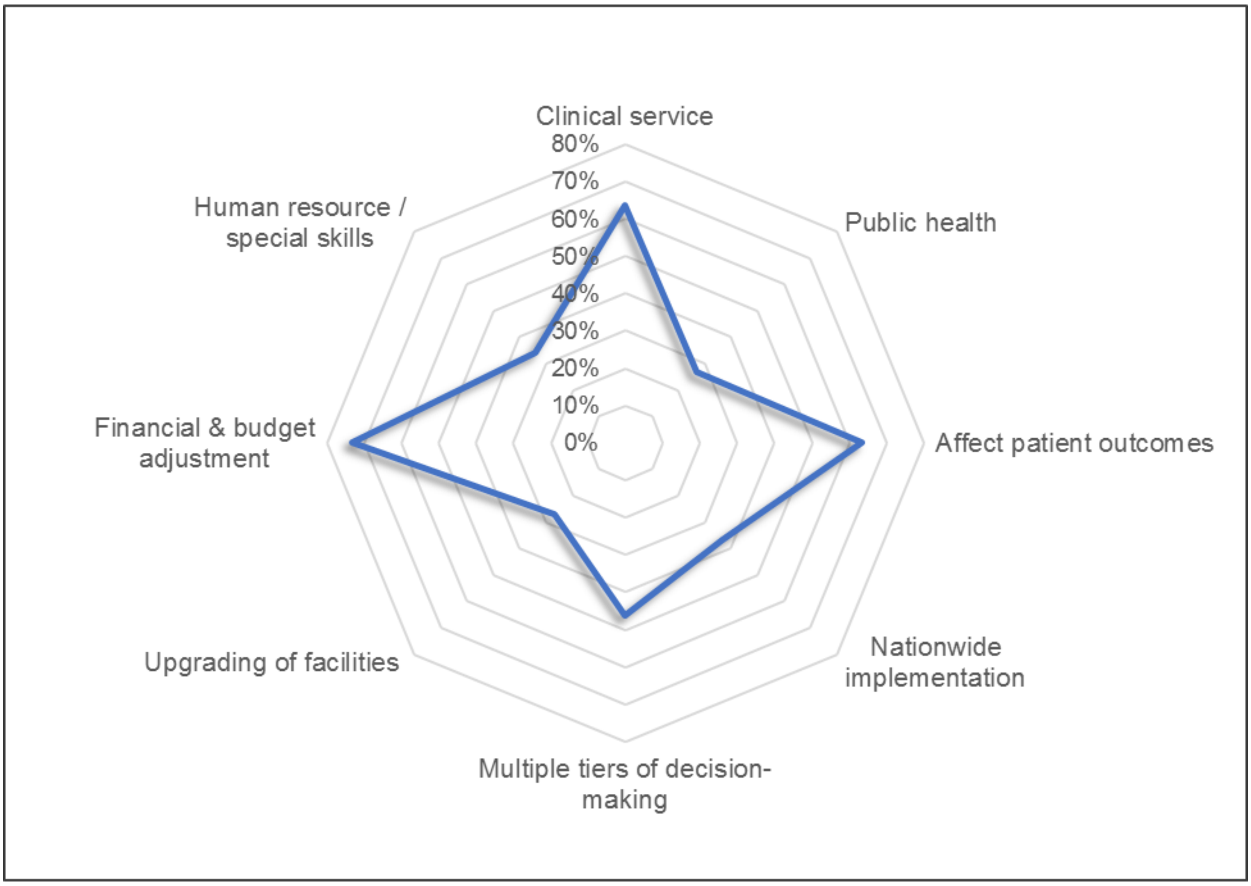

Supplementary 9: Examples of content analysis from respondents' direct quotes on the term 'disinvestment in healthcare'.

<table>
<tr><th>Examples of quotes</th><th>Themes / Sub-themes</th></tr>
<tr><td>"Taking out or withdrawing investment from existing healthcare practices/technology which no longer beneficial and efficient"</td><td rowspan="5">Withdrawing investment or funding

Reduce budget / funding</td></tr>
<tr><td>"Decreasing the budget or funding for health-related programs"</td></tr>
<tr><td>"Stop investment (money, time, human resource) into technologies with low impact or low yield"</td></tr>
<tr><td>"Withdraw an existing investment or reduction of capital expenditures of healthcare thingy including policy, procedures, devices, medicines etc."</td></tr>
<tr><td>"Not investing money, other resources, manpower anymore in healthcare"</td></tr>
<tr><td>"Abstain something that have less value in healthcare system. For example, value of IT devices, health promotion devices."</td><td rowspan="5">Stop practice / provide low-value care (LVC) or inefficient programme

Removal of obsolete technologies</td></tr>
<tr><td>"Stop doing things in healthcare that doesn't benefit anyone"</td></tr>
<tr><td>"Discontinuation of certain healthcare technology/practices/procedures etc due to its devalue in healthcare."</td></tr>
<tr><td>"Termination of unnecessary investment / spending things that does not give a good return in healthcare."</td></tr>
<tr><td>"The necessity to stop offering low-value care and wasteful programs as it not given benefits anymore and furthermore becoming a burden"</td></tr>
<tr><td>"Changes those higher authorities and policy-makers in health need to made to reassess, re-evaluate, re-analyse any form of low impact policies, less accurate guidelines, old technologies, unsuitable drugs, variety of healthcare programmes and others related to healthcare to better and effective ones"</td><td rowspan="4">Process of re-assessment of LVC

Reallocation or shifting of resources</td></tr>
<tr><td>"Reallocation of funding and resources in the healthcare from least effective intervention/treatment based on latest medical evidence to another alternative that provide better outcome to the population."</td></tr>
<tr><td>"Evaluate the allocation provided based on its outcome, to plan either to retain or re-allocate the allocation into other programmes"</td></tr>
<tr><td>"To shift our country's resources from an ineffective healthcare services to a more cost-effective, robust & greater clinical evidence (of practice) ones."</td></tr>
</table>

Supplementary 10: Examples of content analysis from respondents' direct quotes on the need for a formal disinvestment framework in Malaysian healthcare system.

Examples of quotes	Themes / Sub-themes
"Will improve efficiency, reduce redundancies and remove obsolete technologies or work processes."	**Evaluate / monitor previous decision**<br><br>**Healthcare sustainability**
"High cost of healthcare, unnecessary spending, therefore it is needed to ensure the system is more efficient and more people can get care that is both effective and safe"	
"Services / practices should be revised regularly to ensure program efficiency and effectiveness."	
"To identify potential health technologies that warrant reassessment which may have resulted from previous decision-making made in healthcare that were not evidence-based."	
"Save cost and better resource allocations to more important areas as the previous practices might be outdated."	**Priority-based resource allocation process**<br><br>**Transparency in disinvestment decision-making**
"It is not necessarily needed for disinvestment or totally withdrawing the existing budget and resources. But on the other hand, it requires resources to be allocated based on priority and needs of health care practice."	
"So that the decision / evidence can be documented and disseminated formally to all healthcare institutions, can help to standardise practices."	
"Need to have certain criteria before decision on disinvestment is made, as for proper guidance."	
"To provide an evidence-based and structure approach towards disinvestment. Also, it will answer the question on why and how we remove a health technology so that all stakeholders agree with the decision eventually."	
"Improve healthcare delivery and indirectly affect the healthcare practices"	**Improve quality of care and health care services**<br><br>**Shift resources to high-value care**
"Allow better technologies to come in because healthcare needs to be updated according 'what works best at the current time'."	
"This process would allow more latest & relevant healthcare equipment procured periodically to deliver substantial and first-class health care practices."	
"To ensure the optimum level of care and cost-effective intervention is provided to the population."	

Supplementary 11: Subgroup analysis of stakeholders' perspectives on implementing disinvestment initiatives based on years of experience.

a. Experience in current role: 5 years and below (n=62)

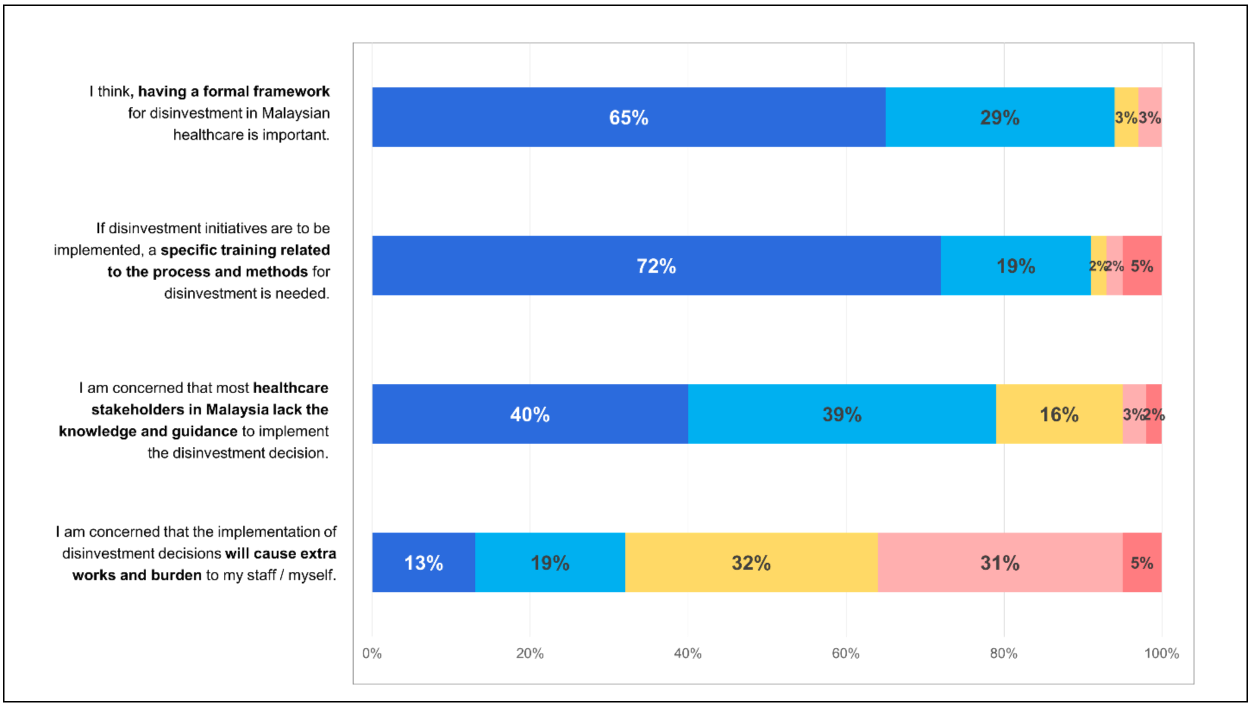

b. Experience in current role: 6-10 years (n=42)

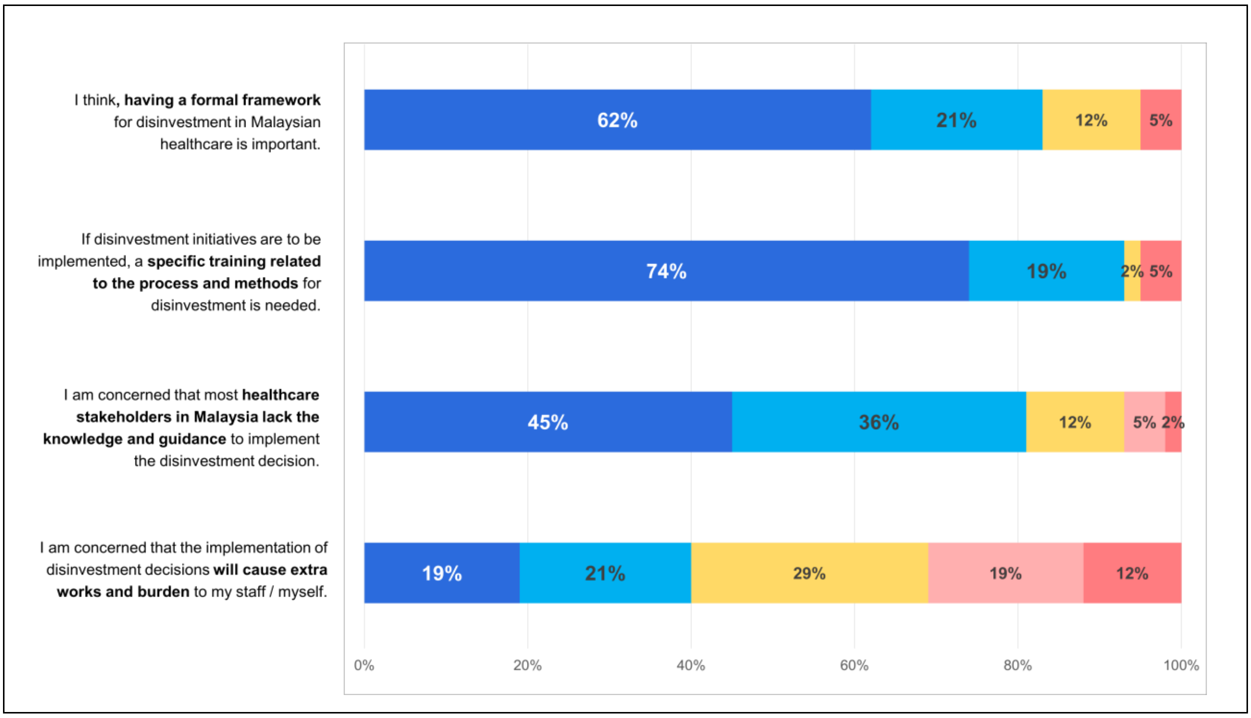

Strongly agree   Somewhat agree   Neither agree nor disagree   Somewhat disagree   Strongly disagree

c. *Experience in current role: More than 10 years (n=49)*

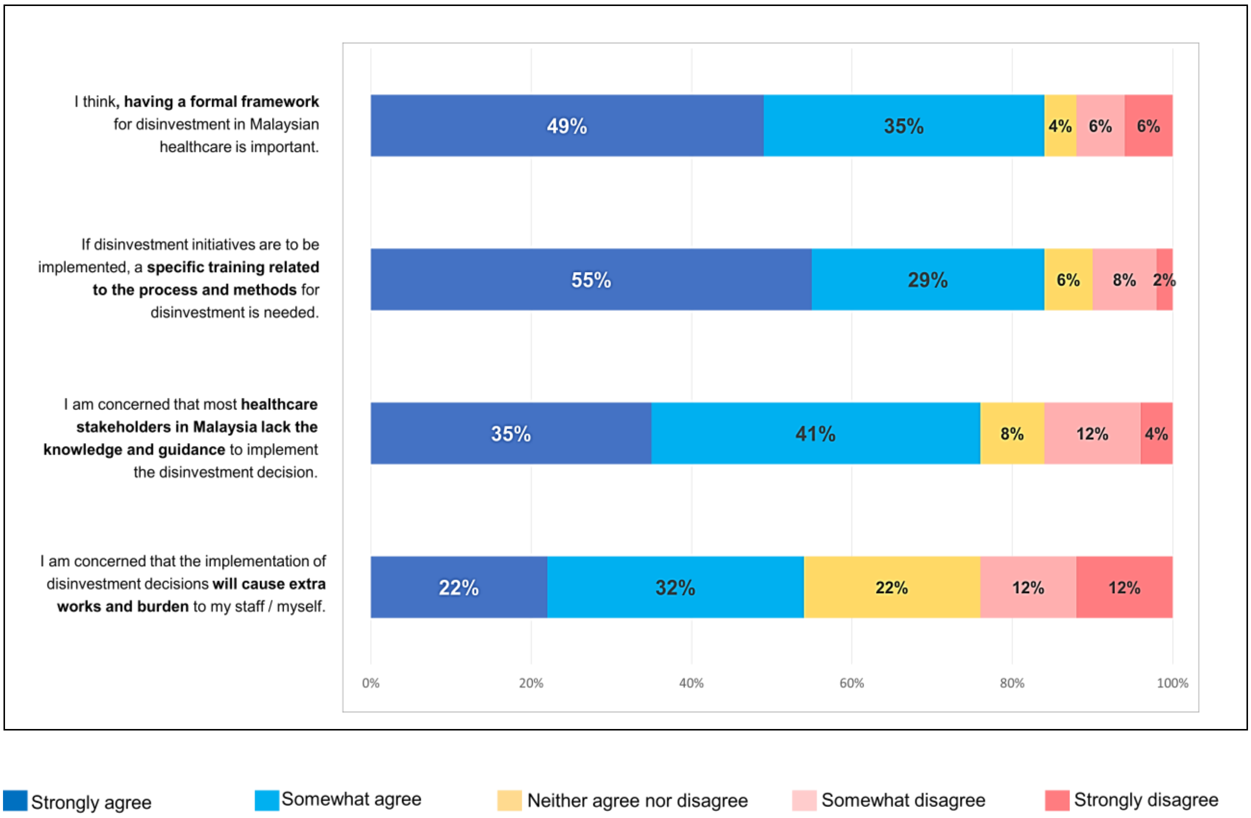

Supplementary 12: Examples of content analysis from respondents' direct quotes on the stakeholders' expectation from the implementation of disinvestment framework in Malaysia.

Examples of quotes	Themes / Sub-themes
"Training of personnel provided, and information dissemination down to staff so we are aware of the objectives and this can align with our mindset and work process."	**Training on implementation of disinvestment framework**
"Awareness and training so that all are informed that the process benefits all level of healthcare. Not only feasible in tertiary hospital but in contrast, cannot be done in district hospital."	
"A clear term of reference (TOR) of the developed framework and relevant training should be offered."	
"A general framework that gives a guideline and links to important queries (legal, ethical and monetary policies)."	
"It should involve all stakeholders to optimize patients' care without compromising the cost of the treatment."	**Stakeholder involvement and awareness**<br>**Health policy development**
"Upper management should be made aware that this is normal and could be done in their lifetime. They should provide support and continuously motivate the staff to change practices."	
"Should be part of the Health White Paper. Not implemented piece-meal. Other parts of the healthcare system need to support implementation. This framework should be briefed to us, hospital administrators."	
"The framework includes opinion from all stakeholders and assessment of pros and cons of the new health technologies versus the older ones."	
"Should be strong policy decision made in consensus of multiple discipline and stakeholders to ensure feasibility of implementation of the framework. Must also ensure that budget to conduct should be allocated."	
"The framework could help in establishing the culture on optimization of technology to facilitate work process."	**Better quality of care & efficient resource allocation**<br>**Transparent and comprehensive process**
"That the implementation will hopefully improve our current healthcare policies without burdening our existing fragile infrastructure."	
"The process should be fair for all low-value care. Thorough but less bureaucratic process to smooth the implementation."	
"It should be transparent and conducted by experts in the field, free of political influence and conflict of interest. The aim should always be for the benefits of the patients and the people."	

Supplementary 13: Subgroup analysis based on stakeholder roles and perspectives on facilitators in implementing disinvestment initiatives (ranking)

<table>
<tr><th rowspan="2">Roles of stakeholders (by group)</th><th colspan="4">Ranking</th></tr>
<tr><th>1st</th><th>2nd</th><th>3rd</th><th>4th</th></tr>
<tr><td>Overall respondents</td><td>Organisational culture</td><td>Stakeholder involvement</td><td>Transparent method</td><td>Integrate local context</td></tr>
<tr><td>Resource allocation decision-makers / budget holders</td><td>Organisational culture</td><td>Transparent method</td><td>Stakeholder involvement</td><td>Integrate local context</td></tr>
<tr><td>Clinical care providers (doctors, pharmacists, nurses, *AHP)</td><td>Transparent method</td><td colspan="2">Organisational culture
Stakeholder involvement</td><td>Integrate local context</td></tr>
<tr><td>Researchers / experts in HTA & health economics, others</td><td>Organisational culture</td><td>Stakeholder involvement</td><td>Transparent method</td><td>Integrate local context</td></tr>
</table>

**AHP, allied health professionals*

Description of facilitators (as in survey questionnaire):

- ***Organisational culture:*** organisational culture for improvement in quality of care and openness to change, including strong leadership.
- ***Stakeholder involvement***: involvement of various stakeholders in healthcare.
- ***Transparent method***: transparent and robust method for identification, prioritisation and assessment of candidates for disinvestment.
- ***Integrate local context:*** integrating local context in formulating recommendation for disinvestment purposes.
